# Incidence, Predictors, and Outcomes of Emergency Surgery Following a Return Visit to the Emergency Department

**DOI:** 10.1016/j.acepjo.2025.100260

**Published:** 2025-10-01

**Authors:** Tzung-Hsin Chou, Jiann-Hwa Chen, Hsiao-Chia Wang, Jun-Wan Gao, Cheng-Chung Fang, Chien-Hua Huang, Chu-Lin Tsai

**Affiliations:** 1 Department of Emergency Medicine, National Taiwan University Hospital, Taipei City, Taiwan; 2 Department of Emergency Medicine, College of Medicine, National Taiwan University, Taipei City, Taiwan; 3 Department of Emergency Medicine, Cathay General Hospital, Taipei City, Taiwan; 4 School of Medicine, Fu Jen Catholic University, New Taipei City, Taiwan

**Keywords:** emergency department, revisit, surgery, outcomes

## Abstract

**Objectives:**

Although return visits to the emergency department (ED) are well-studied, little is known about surgeries following these visits. We aimed to (1) estimate the incidence of surgery after a return ED visit, (2) identify factors associated with these events, and (3) compare outcomes between patients who received surgery after a return visit (revisit surgery) and those who underwent surgery during the initial visit (direct surgery).

**Methods:**

This retrospective cohort study analyzed 454,330 adult ED visits at a tertiary medical center in Taiwan (2016-2019). We identified surgeries performed within 72 hours of an index ED visit and used logistic regression to analyze factors associated with these events. Inpatient outcomes, including mortality and hospital length of stay, were compared between revisit and direct surgery groups using logistic and median regression.

**Results:**

Of 454,330 visits, 4605 (1.0%) involved direct surgery during the initial visit. Among the 403,833 discharged patients, 16,776 (4.2%) returned to the ED within 3 days, and 196 (1.1%) underwent emergent surgery. Factors associated with revisit surgery included triage level, male sex, abdominal pain, older physician age, summer season, and time of presentation. Revisit surgery patients had similar inpatient mortality (adjusted odds ratio, 0.37; 95% CI, 0.09-1.54) and length of stay (adjusted difference, −0.38 days; 95% CI, −1.05 to 0.30) compared with the direct surgery group.

**Conclusions:**

A small fraction (0.05%) of discharged patients required emergent surgery upon return. Identifying risk factors may help target at-risk populations, and outcomes did not differ significantly between revisit and direct surgery groups.

**The Bottom Line**

This study examines the occurrence of emergency surgeries following return visits to the emergency department (ED). Among over 450,000 ED visits, 16,776 patients returned within 3 days, and 196 (0.05%) required emergency surgery. Key risk factors for needing surgery upon return included triage level, abdominal pain, male sex, and older physician age. The study found no significant differences in inpatient outcomes, such as mortality or hospital stay length, between patients who underwent surgery during their initial visit and those who required surgery after returning. Identifying at-risk patients could improve targeted interventions for these cases.

## 1 Introduction

### 1.1 Background

After receiving initial care in the emergency department (ED), most patients are discharged home. However, approximately 5% to 10% return to the ED within 3 days.^1^, ^2^, ^3^ Among the patients returning to the ED, some experience adverse outcomes, such as death in the ED or intensive care unit admission.^4^ These return visits are not only potentially unsafe but also costly, with 1 study showing that the total cost of return visits exceeds the cost of all initial visits combined.^1^ Due to their clinical and financial implications, return ED visit rates are often used as a measure of ED care quality during the initial visit.^5^^,^^6^

### 1.2 Importance

Although much has been written about ED revisits, to our knowledge, few studies have focused on emergency surgeries following such visits. Common scenarios include missed initial diagnoses requiring surgical intervention, such as acute abdominal conditions or fractures.^7^ In addition to ED care-related issues, factors such as patient preferences, shared decision-making, and the natural progression of diseases may also contribute to return visits.^8^, ^9^, ^10^ As a quality metric, ED revisits requiring surgery may reflect a mix of suboptimal care and other factors unrelated to clinical performance, such as patient, illness, or system-related factors. Recent research has suggested focusing on patient outcomes after return visits as a more meaningful quality metric,^11^^,^^12^ such as inpatient outcomes following return surgery. If outcomes for patients undergoing surgery after a return visit are comparable to those for patients receiving surgery during the initial visit, the assumption of delayed or poor care at the initial visit may not hold. However, to our knowledge, no studies have yet explored these follow-up outcomes after return surgery.

### 1.3 Goals of This Investigation

This study aimed to fill these knowledge gaps by (1) estimating the incidence of surgery following a return ED visit, (2) identifying factors associated with these adverse events, and (3) comparing the characteristics and outcomes of patients undergoing surgery after a return ED visit (revisit surgery) with those who received surgery during their initial ED visit (direct surgery).

## 2 Methods

### 2.1 Study Design and Setting

We conducted a retrospective cohort study using electronic health record data from the Cathay General Hospital (CGH) System. The system included a main hospital and 2 branch hospitals. The CGH main hospital is a tertiary academic medical center with 800 beds and approximately 60,000 ED visits per year, with 2 branch regional hospitals collectively serving approximately 100,000 ED visits. This database serves as an integrated clinical data warehouse for all electronic health records in the healthcare system, encompassing inpatient, outpatient, and ED records. The electronic database contains a wealth of information, including demographics, diagnosis, treatment, imaging, laboratory, prescription, nursing, billing, and administrative data. The database is maintained and regularly updated by dedicated research personnel.

For the current study, we retrieved 4 years of deidentified data between January 1, 2016, and December 31, 2019. All CGH data are deidentified but contain a unique, encrypted personal identifier, enabling researchers to link visit records. This study was approved by the CGH Institutional Review Board, which waived the requirement for patient-informed consent. The study was presented following the Strengthening the Reporting of Observational Studies in Epidemiology guidelines (Supplementary Appendix 1).

### 2.2 Study Population

Data from 454,330 adult ED visits (aged 18 and older) were electronically extracted over the 4-year period. The index ED visits were the initial “treat-and-release” ED visits. These index ED visits gave rise to subsequent ED revisits and possible revisit surgeries. A return ED visit was defined as an ED revisit within 72 hours of the index visit. For multiple revisits within 72 hours, we only selected the first revisit.

**Figure 1:**
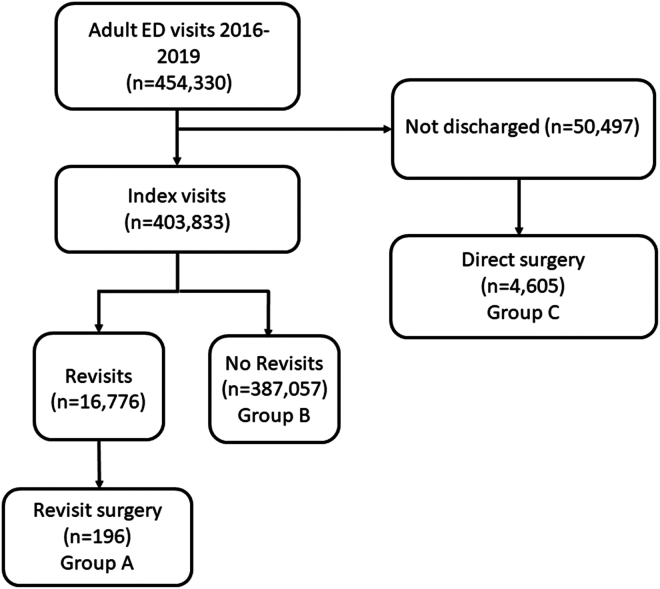
Flow diagram of the patient selection process. ED, emergency department.

The cohort was divided into 2 surgery groups depending on the timing of surgery: (1) direct surgery, ie, patients who were admitted to the hospital and received surgery during the index visit, and (2) revisit surgery, ie, those who were discharged from the ED at the index visit and receive emergency surgery within 24 hours of the return to the ED. The subject selection process is illustrated in Figure 1.

### 2.3 Variables

Patient demographics and clinical information with timestamps in the ED were extracted, including chief complaints on ED presentation, transfer status, mode of arrival, and triage vital signs. Emergency department shifts were classified as day (07:00 am-2:59 pm), evening (3:00 pm-10:59 pm), and night (11:00 pm-06:59 am) shifts. We extracted the 5-level computerized Taiwan Triage and Acuity Scale (TTAS) data, which is a decision-path software comprising a total of 179 structured chief complaints. Based on the TTAS computerized algorithms, patients are prioritized in the following order of acuity: level 1, resuscitation; level 2, emergent; level 3, urgent; level 4, less urgent; and level 5, nonurgent. The TTAS was adapted from the Canadian Triage and Acuity Scale and has been validated against hospitalization, ED length of stay, and resource use.^13^ Major disease diagnoses, such as cancer, end-stage renal disease, and ventilator dependence, were defined by the National Health Insurance in Taiwan (Table S1). This designation represented major comorbidities and was used for risk adjustment in multivariable analysis. The age and sex of the treating physicians were also extracted.

The data extraction was performed by hospital information technology engineers who were blinded to the study hypothesis. The data underwent electronic cleaning, and invalid data were set to missing values after periodic discussions at investigator meetings.

### 2.4 Outcome Measures

The primary outcome measure was the revisit surgery rate, calculated by dividing the number of revisits receiving emergency surgery by the total number of discharged index ED visits. The secondary outcome measures for the 2 surgery groups were inpatient mortality and length of hospital stay. We also examined the most common diagnoses among the 2 surgery groups.

### 2.5 Statistical Analysis

Summary statistics are displayed as proportions (with 95% CIs), means (with SDs), or medians (with IQRs). Patient characteristics were compared between the groups. Univariate associations were examined using Student’s *t* tests, Mann-Whitney tests, and chi-square tests, as appropriate. For the discharged index visits, a multivariable logistic regression was built to investigate the factors associated with revisit surgery. Variables associated with the study outcome in univariate analyses were considered for inclusion in the multivariable model. For the 2 surgery groups, the inpatient outcomes (mortality) and resource use (length of stay) were compared. Multivariable logistic and median (quantile) regression models were used to adjust for differences in patient mix, respectively. Subgroup analysis was also performed to control for differences in diagnosis mix. All odds ratios (ORs) and beta-coefficients are presented with 95% CIs. All analyses were performed using Stata 16.0 software (StataCorp). All *P* values are 2-sided, with *P* < .05 considered statistically significant. The only exception was that for the pairwise comparisons between 3 groups, a Bonferroni-corrected *P* value threshold of .05/3  = .017 was used for statistical significance.

## 3 Results

From January 2016 to December 2019, there were 454,330 adult ED visits (Fig. 1). After excluding those who were not discharged, there were 403,833 index ED visits. There were 16,776 patients who returned to the ED within 72 hours, 196 of whom received emergency surgery (group A, revisit surgery). The overall revisit rate was 4.2%, whereas the rate of revisit surgery was 0.05%. On the other hand, among those who were hospitalized during the first visit, 4605 received surgery (group C, direct surgery). A total of 770 visits were second or higher-order revisits within 72 hours. Of these, none were hospitalized and received emergent surgery.

Table 1 shows the baseline clinical characteristics of the ED patients by revisit status and surgery timing. The study included 196 patients in the revisit surgery group (group A), 387,057 in the no-revisit group (group B), and 4605 in the direct surgery group (group C). The mean age was similar across the groups: 49.9 years in group A, 48.8 years in group B, and 51.1 years in group C. The proportion of female patients was significantly lower in group A (45.4%) compared with group B (54.8%, *P* = .008) but was not significantly different from group C (50.2%, *P* = .19). Regarding the seasonal and presentation time, the distribution of revisit surgery cases across seasons was not significantly different compared with either group B (*P* = .186) or group C (*P* = .226). However, revisit surgery cases were more likely to present between 7:00 am and 2:59 pm (45.4%) compared with group B (35.8%, *P* < .001) and less likely to present during the same time frame compared with group C (52.2%, *P* = .006).

**Table 1:** Baseline clinical characteristics of emergency department patients by revisit status and surgery timing. The characteristics for group A refer to the measurements at the index visit.

Variable	Group A (revisit surgery) N = 196	Group B (no revisit) N = 387,057	*P* value (A versus B)	Group C (direct surgery) N = 4605	*P* value (A versus C)
Age, mean (SD), year	49.9 (19.3)	48.8 (20.2)	.407	51.1 (19.4)	.420
Female sex, n (%)	89 (45.4)	212,143 (54.8)	.008	2312 (50.2)	.190
Season, n (%)			.186		.226
Spring (March-May)	41 (20.9)	96,967 (25.1)		1108 (24.1)	
Summer (June-August)	62 (31.6)	97,383 (25.2)		1183 (25.7)	
Fall (September-November)	44 (22.5)	91,560 (23.7)		1201 (26.1)	
Winter (December-February)	49 (25.0)	101,147 (26.1)		1113 (24.2)	
Presenting time, n (%)			<.001		.006
7:00 am-2:59 pm	89 (45.4)	138,446 (35.8)		2402 (52.2)	
3:00 pm-10:59 pm	47 (24.0)	165,110 (42.7)		1231 (26.7)	
11:00 pm-6:59 am	60 (30.6)	83,501 (21.6)		972 (21.1)	
Subdivision			<.001		.005
Medicine	99 (50.5)	240,694 (62.2)		1794 (39.0)	
Injury	86 (43.9)	133,016 (34.4)		2564 (55.7)	
Ob/Gyn	11 (5.6)	6414 (1.7)		217 (4.7)	
Other	0 (0.0)	6933 (1.8)		30 (0.7)	
Major disease, n (%)	12 (6.1)	27,522 (7.1)	.591	280 (6.1)	.981
Arrival by ambulance, n (%)	15 (7.7)	38,205 (9.9)	.298	1493 (32.4)	<.001
Most common chief complaint, n (%)			<.001		.347
Abdominal pain	53 (27.0)	37,493 (9.7)		991 (21.5)	
Dizziness	0 (0.0)	17,123 (4.4)		15 (0.3)	
Chest pain	1 (0.5)	11,738 (3.0)		34 (0.7)	
Fever	2 (1.0)	14,603 (3.8)		33 (0.7)	
Dyspnea	0 (0.0)	5478 (1.4)		34 (0.7)	
Other	140 (71.4)	300,622 (77.7)		3498 (76.0)	
Triage level, n (%)			<.001		.016
1	4 (2.0)	4868 (1.3)		253 (5.5)	
2	33 (16.8)	42,470 (11.0)		942 (20.5)	
3	156 (79.6)	294,655 (76.1)		3388 (73.6)	
4 + 5	3 (1.5)	45,064 (11.6)		22 (0.5)	
Vital sign at triage					
Systolic blood pressure, mean (SD), mm Hg	134.4 (27.1)	134.7 (25.7)	.894	136.9 (28.5)	.213
Heart rate, mean (SD), beats per min	86.7 (17.2)	87.5 (18.6)	.522	86.1 (17.9)	.640
Body temperature, mean (SD), °C	37.0 (0.7)	36.9 (0.8)	.336	36.9 (0.7)	.220
Respiratory rate, mean (SD), breaths per min	18.3 (1.5)	18.3 (2.4)	.757	18.3 (1.7)	.900
Oxygen saturation, median (IQR), %	98 (97-99)	98 (97-99)	.800	98 (97-99)	.878
ED length of stay, median (IQR), h	3 (2-7)	1 (1-3)	<.001	3 (2-5)	<.001
Age of treating physician, mean (SD), y	37.8 (8.1)	36.2 (7.3)	.006	37.5 (7.5)	.651
Sex of treating physician, n (%)			.893		.235
Male	168 (85.7)	333,058 (86.1)		4075 (88.5)	
Female	28 (14.3)	53,999 (14.0)		530 (11.5)	
Discharged on weekends	65 (33.2)	109,642 (28.3)	.133		

ED, emergency department.

With respect to reasons for the ED visit, for the 2 surgery groups, revisit surgery cases were more likely to visit due to medical problems for the first time (50.5%) compared with group C (39.0%, *P* = .005). Group C patients were more likely to arrive at the ED by ambulance than group A patients. Abdominal pain was the most common chief complaint among revisit surgery cases (27.0%), significantly higher compared with group B (9.7%, *P* < .001) but similar to group C (21.5%, *P* = .347). Patients in group A were less likely to be triaged at lower levels (4 + 5) (1.5%) compared with group B (11.6%, *P* < .001) but more likely compared with group C (0.5%, *P* = .016). Vital signs at triage, such as systolic blood pressure, heart rate, body temperature, and respiratory rate, were comparable across the groups (*P* > .2 for all comparisons).

Regarding the ED stay and physician characteristics, the median ED length of stay was longer for revisit surgery cases (3 hours; IQR, 2–7) compared with group B (1 hour; IQR, 1-3, *P* < .001). The mean age of the treating physician was slightly higher for group A (37.8 years) compared with group B (36.2 years, *P* = .006) but not significantly different from group C (37.5 years, *P* = .651). There were no differences in the sex of the treating physician between the groups.

In the multivariable analysis adjusting for potential confounders (Table 2), factors significantly associated with revisit surgery included female sex (adjusted odds ratio [aOR], 0.58; 95% CI, 0.43-0.78; *P* < .001), summer season (aOR, 1.50; 95% CI, 1.03-2.30; *P* = .037), presenting time between 3:00 pm and 10:59 pm (aOR, 0.44; 95% CI, 0.30-0.63; *P* < .001), and abdominal pain as the chief complaint (aOR, 3.62; 95% CI, 2.59-5.05; *P* < .001). Lower triage levels (4 + 5) were associated with significantly lower odds of revisit surgery (aOR, 0.11; 95% CI, 0.03-0.44; *P* = 0.002). A 10-year increase in the age of the treating physician was associated with higher odds of revisit surgery (aOR, 1.35; 95% CI, 1.12-1.64; *P* = 0.002).

**Table 2:** Multivariable model of factors associated with emergency department revisit surgery.

Variable	Adjusted odds ratio	95% CI	*P* value
Female sex	0.58	0.43-0.78	<.001^a^
Season			
Spring (March-May)	1.00		
Summer (June-Aug.)	1.50	1.03-2.3	.037^a^
Fall (September-November)	1.19	0.76-1.86	.435
Winter (December-Feburuary)	1.26	0.82-1.95	.290
Presenting time			
7:00 am-2:59 pm (reference)	1.00		
3:00 pm-10:59 pm	0.44	0.30-0.63	<.001^a^
11:00 pm-6:59 am	0.93	0.65-1.33	.686
Abdominal pain as the chief complaint	3.62	2.59-5.05	<.001^a^
Triage level			
1	1.75	0.54-5.63	.349
2	1.35	0.88-2.07	.176
3 (reference)	1.00		
4 + 5	0.11	0.03-0.44	.002^a^
Age of treating physician, per 10-y increase	1.35	1.12-1.64	.002^a^

In addition to variables in the table, the model adjusted for major disease, body temperature, heart rate, respiratory rate, systolic blood pressure, patient age, arrival by ambulance, emergency department length of stay, physician sex, and discharge on the weekend. Statistically nonsignificant predictors are not shown in the table.

a Significant results.

The distribution of operative diagnoses varied between the direct surgery group and the revisit surgery group (Table 3). In the direct surgery group, fractures were the most common diagnosis in the direct surgery group, comprising 28.9% of cases, followed by appendicitis (20.9%) and intracranial hemorrhage (5.7%). Additional diagnoses in this group included cholecystitis (4.6%), cesarean section (4.0%), and urolithiasis (2.0%). In contrast, among revisit surgery cases, the most common operative diagnosis was appendicitis, accounting for 36.2% of cases, followed by cesarean section (11.7%) and fractures (9.7%). Other notable diagnoses included urolithiasis (8.7%) and intracranial hemorrhage (4.6%). Although rare, hollow organ perforation was more prevalent (2.6%) in the revisit surgery group than in the direct surgery group (1.3%). A detailed list for the 2 surgery groups is included in the Table S2.

**Table 3:** Most common operative diagnoses by surgery timing.

Direct surgery (n = 4605)		Revisit surgery (n = 196)	
Operative diagnosis, n (%)		Operative diagnosis, n (%)	
Fracture	1332 (28.9)	Appendicitis	71 (36.2)
Appendicitis	962 (20.9)	Cesarean section	23 (11.7)
Intracranial hemorrhage	264 (5.7)	Fracture	19 (9.7)
Cholecystitis	214 (4.6)	Urolithiasis	17 (8.7)
Cesarean section	183 (4.0)	Intracranial hemorrhage	9 (4.6)
Urolithiasis	91 (2.0)	Hollow organ perforation	5 (2.6)
Ectopic pregnancy	89 (1.9)		
Hollow organ perforation	59 (1.3)		

Regarding the inpatient outcomes after surgery (Table 4), the inpatient mortality was observed in 1.0% of revisit surgery cases (group A) and 2.5% of direct surgery cases (group C). Although the mortality rate was lower in group A, the difference was not statistically significant (*P* = .180). After adjusting for age and sex, the odds of inpatient mortality for group A compared with group C remained nonsignificant (aOR, 0.37; 95% CI, 0.09-1.54; *P* = .174). The median hospital length of stay was 4 days (IQR, 2-5) for group A and 4 days (IQR, 2-7) for group C. The difference in hospital length of stay was not statistically significant after adjustment (adjusted difference in medians: −0.38 day; 95% CI, −1.05 to 0.30; *P* = 0.271). Subgroup analyses in patients with appendicitis or intracranial hemorrhage also showed no statistical differences in inpatient mortality and hospital length of stay (Table S3).

**Table 4:** Study outcomes by surgery timing.

Variable	Group A (revisit surgery) N = 196	Group C (direct surgery) N = 4605	*P* value (A vs C)	Adjusted OR or beta coefficient (95% CI) for group A (vs group C)^a^
Inpatient mortality, n (%)	2 (1.0)	117 (2.5)	.180	0.37 (0.09-1.54) *P* = 0.174
Hospital length of stay, median (IQR), d	4 (2-5)	4 (2-7)	<.001	−0.38 (−1.05 to 0.30) *P* = 0.271

OR, odds ratio.

a Adjusted for age and sex.

## 4 Limitations

This study has some potential limitations. First, this was a single-center study at a tertiary medical center, and our findings may not be generalizable to hospitals in different settings. Second, because this was a retrospective study, we did not have detailed information on the treating physician’s decision-making process or patients’ preferences. In addition, physician experience and specialty information are not available. These factors may also impact the rate of revisit surgery and patient outcomes. The factors associated with revisit surgery identified in this study were hypothesis-generating in nature, and these associations need to be tested in future studies. Third, not all cases of revisit surgery necessarily represent missed or delayed diagnoses. Some patients may have had evolving clinical conditions or were appropriately managed at the initial ED visit. In-depth case reviews would be better suited to address this type of question (appropriateness or preventability). Finally, despite a large sample, the number of inpatient deaths was small, resulting in reduced statistical power to detect differences in groups. Furthermore, heterogeneity in the comparison groups was high, and we attempted to mitigate this by subgroup analysis. A larger multicenter study is warranted to validate our findings and investigate a wider range of patient outcomes.

## 5 Discussion

In this large database analysis of 454,330 emergency department (ED) visits, we found that a very small fraction of discharges (0.05%) required emergent surgery during return visits. This large sample allowed us to identify patient and physician factors associated with this at-risk population. Nonetheless, patients undergoing surgery after a return ED visit did not demonstrate worse outcomes compared with those who had surgery during their initial visit.

The overall rate of emergent surgery during return ED visits was 0.05%. In contrast, a previous smaller study of nontraumatic adult patients reported a higher rate of 0.2%,^14^ likely due to the exclusion of trauma cases, which generally have lower revisit and admission rates.^15^ In this study, among patients undergoing surgery during a revisit, the 3 most common diagnoses were acute appendicitis (14.3%), malignancy-related complications (12.6%, including bleeding or intestinal obstruction), and cholecystitis (10.9%).^14^ These findings align with ours and were probably a subset of the conditions reported in our study.

We identified several factors associated with revisit surgery, with abdominal pain being a prominent example. Patients presenting with abdominal pain had a 3.62-fold increased likelihood of requiring surgery upon revisiting. In an Italian ED study, acute abdominal pain (AAP) accounted for 5.76% of total ED visits.^16^ Patients with abdominal pain often present with ambiguous symptoms and are frequently discharged with a diagnosis of nonspecific abdominal pain (NSAP),^17^ This study found that NSAP and renal colic together represented over 60% of all AAP cases.^16^ Similarly, a Scandinavian study spanning 26 years found that the most common causes of AAP were NSAP (31% to 37%), followed by acute appendicitis (11% to 23%), biliary disease (9% to 11%), and bowel obstruction (5% to 7%).^18^ In our revisit study, appendicitis was the most prevalent diagnosis associated with revisit surgery (36.2%), suggesting its diagnostic challenge during the first visit.

NSAP often evolved into more specific diagnoses upon revisit, implicating diagnostic errors. Among abdominal pain-related revisit admissions, diagnostic errors were observed in 35 out of 100 cases.^19^ A US study of 1000 consecutive AAP cases over 35 years reported that, despite a 6-fold increase in computed tomography use over time, 2 cases of missed surgical disease were still identified in 2007.^20^ To address this patient safety issue, a study proposed “symptom-disease dyad electronic triggers” to flag patient records with a high likelihood of diagnostic error, such as ED visits for abdominal pain followed by diagnoses of appendicitis or perforated diverticulitis within 10 days.^21^ A study proposed strategies for reducing diagnostic errors in abdominal pain, including thorough history-taking, ordering adequate tests, and ensuring follow-up of abnormal results.^19^ Although rare (2.6% in our study), serious missed diagnoses, such as hollow organ perforation in cases of blunt abdominal trauma or nontraumatic abdominal pain,^22^^,^^23^ can be life-threatening and result in complications like septic shock.

In our study, fractures were the most common diagnosis for direct surgery at initial visits and accounted for nearly 10% of revisit surgeries. A study found that 1.1% of fractures were missed during initial evaluations, with pelvis and hip fractures in older adults being the most frequently overlooked.^24^ Patients required surgery in 9.3% of missed fractures.^24^ Intracranial hemorrhages (ICH) were another significant concern, often associated with serious neurologic conditions or malpractice claims. For example, 0.5% of headache patients returned to the ED with a serious neurologic condition, including ICH, or died in-hospital within 30 days.^25^

In our study, male patients were at higher risk for revisit surgery, particularly for appendicitis, urolithiasis, and fractures. Men consistently exhibited a higher prevalence of appendicitis and urolithiasis across all age groups, with a 2 to 2.8-fold increased risk compared with women.^26^, ^27^, ^28^ Summer was also identified as a risk factor for revisit surgery, potentially due to seasonal increases in appendicitis and urolithiasis related to dehydration.^26^^,^^29^ The male-to-female ratio for joint fractures was also approximately 2 to 1.^30^

With respect to physician age, it is somewhat concerning that older age was associated with an increased risk of revisit surgery. A recent Medicare study reported that patients treated by emergency physicians under 40 years old had lower 7-day mortality rates compared with those treated by physicians aged 50 or older.^31^ Although it may be tempting to attribute this difference to older physicians being less up-to-date with current medical knowledge, alternative explanations should be considered. Older physicians may have a greater tolerance for risk and higher discharge rates.^32^^,^^33^ Additionally, some older practicing emergency physicians may lack board certification, suggesting that differences in outcomes could stem from variations in training and specialization rather than age alone.

Our outcome analysis showed that the revisiting group did not have higher in-hospital mortality. In fact, this group had shorter hospital stays and lower inpatient mortality, although statistical significance was limited by the small number of deaths. Notably, more patients receiving direct surgery were triaged as level 1 or 2 (5.5% and 20.5%, respectively) compared with revisit surgery patients (2.0% and 16.8%, respectively). This may suggest that critically ill patients (eg, intracranial hemorrhage, cholecystitis, or ectopic pregnancy) were managed during their first visit, leaving less severe cases for revisit surgeries with slightly better outcomes. Nonetheless, these findings should not deter efforts to improve diagnostic accuracy during the initial ED visit to minimize downstream complications, such as hollow organ perforation, which can lead to poor patient outcomes. In addition, future studies should examine other outcome measures, such as expenditure and patient-reported outcomes, as we only included limited outcomes that may not reflect the entire patient experience.

In summary, in this large study of 454,330 ED visits, we found that only a small fraction of discharges (0.05%) required emergent surgery during return ED visits. Our analysis identified patient and physician factors that could help target this at-risk population. Nonetheless, patients who underwent surgery after a return ED visit did not experience poorer selected outcomes compared with those who had surgery during their initial visit. However, given the occurrence of catastrophes in the revisit surgery group, efforts should focus on reducing diagnostic errors during the initial visit, particularly for cases of nonspecific abdominal pain. Regular reviews of catastrophic revisit surgery cases could provide valuable insights to enhance patient safety and improve outcomes.

## Author Contributions

THC and CLT conceived and designed the study. JHC and HCW obtained the data. JWG and CLT analyzed the data. THC and CLT drafted the manuscript. All authors interpreted the results and contributed substantially to the manuscript revision. CLT supervised the study and take responsibility for the paper as a whole.

## Funding and Support

This project was supported by grants from the Ministry of Science and Technology (112-2314-B-002-264), the 10.13039/501100004737National Health Research Institutes (NHRI-EX114-11332PI), and the 10.13039/501100005762National Taiwan University Hospital (111-CGN-0005 and 113-UN-0017).

## Conflict of Interest

All authors have affirmed they have no conflicts of interest to declare.
