# Supplementary material for: Incidence, Predictors, and Outcomes of Emergency Surgery Following a Return Visit to the Emergency Department
Source: J Am Coll Emerg Physicians Open. 2025 Oct 1;6(6):100260. doi: 10.1016/j.acepjo.2025.100260 (PMC12516037; doi:10.1016/j.acepjo.2025.100260)
Supplement: Supplementary Table 1 [file mmc1.docx]

**Table S1.** A List of major illnesses defined by the National Health Insurance in Taiwan and the percentage of each category.

1. Cancer
2. Hereditary deficiencies of clotting factors
3. Severe hemolytic or aplastic anemia
4. End-stage renal disease on dialysis
5. Systemic autoimmune diseases
6. Chronic psychiatric disorders or dementia
7. Congenital metabolic anomalies
8. Major organ congenital anomalies
9. Massive burn
10. Organ transplantation
11. Cerebral palsy
12. Major trauma rated 16 or above on the severity scale
13. Long-term mechanical ventilation dependency
14. Severe malnutrition on long-term parenteral nutrition
15. Decompression sickness
16. Myasthenia gravis
17. Congenital immunodeficiencies
18. Spinal cord injuries
19. Occupational lung disease
20. Cerebrovascular disease (acute stage)
21. Multiple sclerosis
22. Congenital muscular dystrophy
23. Congenital anomalies of skin
24. Leprosy (Hansen’s disease)
25. Liver cirrhosis with complication
26. Premature infants with complications
27. Toxic effect of arsenic and its compounds (black foot disease)
28. Motor neuron disease
29. Creutzfeldt-Jakob disease
30. Rare disease

| **Disease, n (%)** | **N = 990,896 (all major illness cardholders in Taiwan) in 2024** |
| --- | --- |
| Cancer | 463,269 (46.8) |
| Hereditary deficiencies of clotting factors | 1,856 (0.4) |
| Severe hemolytic or aplastic anemia | 1,475 (0.2) |
| End-stage renal disease on dialysis | 89,721 (9.1) |
| Systemic autoimmune diseases | 139,826 (14.1) |
| Chronic psychiatric disorders or dementia | 189,102 (19.1) |
| Congenital metabolic anomalies | 18,388 (1.9) |
| Major organ congenital anomalies | 37,868 (3.8) |
| Massive burn | 292 (0.0) |
| Organ transplantation | 17,465 (1.8) |
| Cerebral palsy | 13,032 (1.3) |
| Major trauma rated 16 or above on the severity scale | 12,485 (1.3) |
| Long-term mechanical ventilation dependency | 11,196 (1.1) |
| Severe malnutrition on long-term parenteral nutrition | 75 (0.0) |
| Decompression sickness | 10 (0.0) |
| Myasthenia gravis | 6,132 (0.6) |
| Congenital immunodeficiencies | 218 (0.0) |
| Spinal cord injuries | 7,459 (0.8) |
| Occupational lung disease | 1,404 (0.1) |
| Multiple sclerosis | 62 (0.0) |
| Congenital muscular dystrophy | 367 (0.0) |
| Congenital anomalies of skin | 131 (0.0) |
| Leprosy (Hansen’s disease) | 124 (0.0) |
| Liver cirrhosis with complication | 3,602 (0.4) |
| Premature infants with complications | 8 (0.0) |
| Toxic effect of arsenic and its compounds (black foot disease) | 53 (0.0) |
| Motor neuron disease | 96 (0.0) |
| Creutzfeldt-Jakob disease | 20 (0.0) |
| Rare disease | 15,171 (1.5) |
