# Supplementary material for: Incidence, Predictors, and Outcomes of Emergency Surgery Following a Return Visit to the Emergency Department
Source: J Am Coll Emerg Physicians Open. 2025 Oct 1;6(6):100260. doi: 10.1016/j.acepjo.2025.100260 (PMC12516037; doi:10.1016/j.acepjo.2025.100260)
Supplement: Supplementary Table 2 [file mmc2.docx]

**Table S2**. Operative diagnoses and procedures by revisit status.

| **Direct surgery**  **(n= 4,605)** | | **Revisit surgery**  **(n=196)** | |
| --- | --- | --- | --- |
| Operative diagnosis/procedure, n (%) | | Operative diagnosis/procedure, n (%) | |
| Fracture | 1,332 (28.9) | Appendicitis | 71 (36.2) |
| Appendicitis | 962 (20.9) | Cesarean section | 23 (11.7) |
| Intracranial hemorrhage | 264 (5.7) | Fracture | 19 (9.7) |
| Debridement & wound care (various sites: soft tissue, tendon, muscle, fascia, etc) | 258 (5.6) | Urolithiasis | 17 (8.7) |
| Various drainage for intra-abdominal or urinary tract infection (eg, intra-abdominal abscess, biliary tract infection, pyelonephritis) | 247 (5.4) | Various drainage for intra-abdominal or urinary tract infections (eg, intra-abdominal abscess, biliary tract infection, pyelonephritis) | 13 (6.6) |
| Vascular access and interventions (eg, permanent hemodialysis catheter placement, arteriovenous fistula thrombectomy) | 243 (5.3) | General surgical procedures (eg, debridement, incision and drainage) | 12 (6.1) |
| Cholecystitis | 214 (4.6) | Intracranial hemorrhage | 9 (4.6) |
| Cesarean section | 183 (4.0) | Vascular access and interventions (eg, permanent hemodialysis catheter placement, arteriovenous fistula thrombectomy) | 9 (4.6) |
| Urolithiasis | 91 (2.0) | Hollow organ perforation | 5 (2.6) |
| Ectopic pregnancy | 89 (1.9) | Cholecystitis | 4 (2.0) |
| Hollow organ perforation | 59 (1.3) | Ectopic pregnancy | 4 (2.0) |
| Thoracic surgeries (eg, tube thoracostomy, pericardial window, and video-assisted thoracoscopic surgery) | 57 (1.2) | Other orthopedic surgeries (eg, for open or closed reduction of joint dislocation) | 3 (1.5) |
| Colorectal / anorectal surgeries (eg, fistulotomy, hemorrhoidectomy, colon resection and stoma creation) | 55 (1.2) | Thoracic procedures (eg, tube thoracostomy or video-assisted thoracoscopic surgery) | 3 (1.5) |
| Other abdominal surgeries (eg, adhesiolysis for adhesion ileus, herniorrhaphy for hernia) | 52 (1.1) | Spine surgeries (eg, vertebroplasty or epidural abscess) | 2 (1.0) |
| Other urologic procedures (eg, cystostomy, cystoscopy and blood clot evacuation from bladder) | 51 (1.1) | Anorectal procedures (eg, fistulotomy or hemorrhoidectomy) | 2 (1.0) |
| Joint surgeries (arthrodesis, or joint dislocation reduction) | 48 (1.0) |  |  |
| Foreign body removal (eg, intraocular or soft tissue foreign body extraction) | 45 (1.0) |  |  |
| Other gynecologic surgeries (eg, myomectomy, total abdominal hysterectomy, and laparoscopic ovarian cystectomy) | 43 (0.9) |  |  |
| Other obstetric procedures (non Cesarean section) (eg, dilatation and curettage) | 41 (0.9) |  |  |
| Cardiac surgeries (eg, coronary artery bypass grafting) | 37 (0.8) |  |  |
| Spine surgeries (eg, laminectomy, posterior spinal fusion) | 34 (0.7) |  |  |
| Neurovascular interventions (eg, aneurysm clipping) | 33 (0.7) |  |  |
| Tendon/nerve/muscle repair and muscle reconstruction | 31 (0.7) |  |  |
| ENT & airway procedures (eg, tracheostomy) | 29 (0.6) |  |  |
| Ophthalmologic surgeries (eg, vitrectomy, endophotocoagulation) | 27 (0.6) |  |  |
| Plastic & reconstructive surgeries (eg, nail bed or eyelid/brow repair) | 26 (0.6) |  |  |
| Miscellaneous | 23 (0.5) |  |  |
| Burns and skin grafting (eg, full-thickness skin grafts, various flaps | 21 (0.5) |  |  |
| Amputation (eg, above or below knee amputation) | 10 (0.2) |  |  |
