# Supplementary material for: Incidence, Predictors, and Outcomes of Emergency Surgery Following a Return Visit to the Emergency Department
Source: J Am Coll Emerg Physicians Open. 2025 Oct 1;6(6):100260. doi: 10.1016/j.acepjo.2025.100260 (PMC12516037; doi:10.1016/j.acepjo.2025.100260)
Supplement: Supplementary Table 3 [file mmc3.docx]

**Table S3**. Study outcomes by revisit status within subgroups.

| **Variable** | **Group A**  **(revisit surgery)** | **Group C**  **(direct surgery)** | **P value**  **(A vs C)** | **Adjusted OR or beta coefficient (95% CI) for Group A (vs Group C)*** |
| --- | --- | --- | --- | --- |
| *Appendicitis subgroup* | N=71 | N=962 |  |  |
| Inpatient mortality, n (%) | 0 (0.0) | 1 (0.1) | 1.0 | NC |
| Hospital length of stay, median (IQR), day | 3 (2-5) | 3 (2-4) | 0.108 | <0.001 (-0.51 to 0.51)  P = 1.00 |
| *Intracranial hemorrhage subgroup* | N=9 | N=264 |  |  |
| Inpatient mortality, n (%) | 0 (0.0) | 46 (17.4) | 0.365 | NC |
| Hospital length of stay, median (IQR), day | 8 (5-14) | 18 (7-30) | 0.141 | -4.67 (-19.67 to 10.34)  P = 0.541 |

Abbreviations: CI = confidence interval; OR = odds ratio; NC = non-calculable due to zero cell; IQR = interquartile range.

*Adjusted for age and sex.
